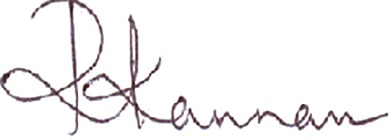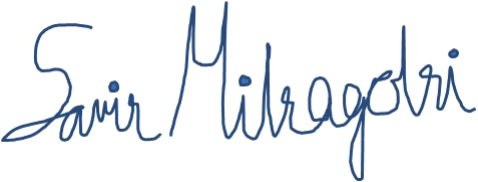# Engineering clinical translation‐Introduction to Special Issue Dedicated to 2017 Bioengineering and Translational Medicine Conference

**DOI:** 10.1002/btm2.10115

**Published:** 2018-10-08

**Authors:** Rangaramanujam M. Kannan, Samir Mitragotri

**Affiliations:** ^1^ John Hopkins University, School of Medicine; ^2^ Harvard University

1

A timely convergence of many factors, including gene‐editing technologies, machine learning, nanomedicine, and artificial intelligence, is making it tremendously exciting to be an engineer at the interface of medicine. This is further fueled by the recognition that we need bold strategies to tackle health care challenges across the world including ways to prevent autism and Alzheimer's, making medicines economical, and speeding up the process of translation. Brining perspectives from across the spectrum of stakeholders is critical to speeding up translation. Engineers are well‐positioned to be an enabling community in this regard. Recognizing this opportunity, the American Institute of Chemical Engineers (AIChE) and Society of Biological Engineers (SBE) launched this journal and launched the conference series starting in 2016. The second conference in this series was held in Minneapolis on the heels of the annual AIChE meeting from October 28‐29, 2017, featuring exciting talks and posters covering focus areas such as stem cells/regenerative medicine, immunoengineering, regulatory aspects, biopharmaceuticals, and gene/drug delivery. These are being highlighted in this second issue highlighting conference papers, edited by Rangaramanujam Kannan (Johns Hopkins Medicine) and Samir Mitragotri (Harvard).

*Couvas and Prausnitz* describe the development of novel ways to deliver adjuvant‐free hepatitis B vaccine through the skin in a simple manner through microneedle patches, which could help fight global childhood infections. Genetically engineering bacteria for superior localized therapies was a key theme of the conference. *McCay and Bentley* illustrate the potential of this approach in Crohn's Disease by engineering a “smart” probiotics platform, where *Escherichia coli* selectively synthesize and secrete biotherapeutics in the presence of nitric oxide, an intestinal biomarker for Crohn's disease. *Geldart and Kaznessis* showing promising proof of concept results of engineering *E. coli* that can simultaneously produce and secrete three antimicrobial peptides to specifically target and kill Enterococcus in the intestine and prevent their translocation into the blood. The simultaneous expression of the engineered peptides not only inhibited the bacteria but also prevented the development of resistance. *Kumar and Pillay* describe the utility of in silico analytico‐mathematical interpretation and description of biopolymeric assemblies, with implications in their use in biomedical applications.

Regenerative medicine was a key theme in the conference, with multiple articles featured on this theme in both special issues. *Willadsen and Parashurama* contribute an extensive, forward looking review on the benefits of emerging molecular imaging strategies that can provide informativeand personalized ways for regenerative medicine to make the next strides towards translation. *Tseropoulos and Andreadis* emphasize the importance of precise methods for the production of stem cells by describing well‐defined method for the derivatization of human neural crest and keratinocyte cells. The special issue is rounded out by the contribution of *Shahvari and Matthew* who address strategies for improving the scalability and the stability of mesenchymal stem cells (MSCs), suggesting electrospraying as a potentially efficient, versatile, and scalable approach for the encapsulation and preservation of MSCs for subsequent use in bioprinting and regenerative medicine.

In many ways, the articles in both of the two conference special issues, and the BTM conference highlighted the rich variety and depth of perspectives bioengineers can bring to important health issues, through their collaborations with physicians, regulators, and investors. We may be at the tipping point where such perspectives can accelerate translation of discoveries, human impacting health meaningfully.